# Increased plasma levels of the soluble Mer tyrosine kinase receptor in systemic lupus erythematosus relate to disease activity and nephritis

**DOI:** 10.1186/ar3316

**Published:** 2011-04-15

**Authors:** Jun Wu, Carl Ekman, Andreas Jönsen, Gunnar Sturfelt, Anders A Bengtsson, Anders Gottsäter, Bengt Lindblad, Elisabet Lindqvist, Tore Saxne, Björn Dahlbäck

**Affiliations:** 1 Department of Laboratory Medicine, Section of Clinical Chemistry, Lund University, Wallenberg Laboratory, Skåne University Hospital, Södra Förstadsgatan 101, Malmö, SE 205 02, Sweden; 2 Department of Clinical Sciences, Section of Rheumatology, Lund University, Skåne University Hospital, Getingevägen 4, Lund, SE 22185, Sweden; 3 Department of Clinical Sciences, Vascular Center, Lund University, Skåne University Hospital, Södra Förstadsgatan 101, Malmö, SE 205 02, Sweden; 4 Department of Laboratory Medicine, People's Hospital, Peking University, No.11 Xizhimen South Street, Beijing, 100044, PR China

## Abstract

**Introduction:**

Mer and Tyro3 are receptor tyrosine kinases important for the phagocytosis of apoptotic cells. Together with Axl, they constitute the TAM receptor family. These receptors can be shed from the cell membrane and their soluble extracellular regions can be found in plasma. The objective of this study was to elucidate whether the plasma levels of soluble Mer (sMer) and Tyro3 (sTyro3) were increased in systemic lupus erythematosis (SLE), rheumatoid arthritis (RA), or critical limb ischemia (CLI).

**Methods:**

ELISA kits were used to test plasma concentrations in controls and in patients with SLE, RA or CLI.

**Results:**

Increased levels of, in particular, sMer and, to some extent, sTyro3, were found in patients with SLE or RA, but not in patients with CLI. Patients with SLE demonstrated the highest sMer levels and there was a strong correlation to higher SLE disease activity score (SLEDAI). In contrast, in patients with RA, the sMer levels did not correlate with the disease activity score (DAS). In SLE, sMer levels were particularly high in those with lupus nephritis, patients who also had decreased C1q levels and increased titers of anti-DNA antibodies. After therapy, the plasma concentrations of sMer decreased in parallel to the decrease in SLEDAI score.

**Conclusions:**

The plasma concentrations of sMer and sTyro3 were significantly increased in patients with active SLE and RA, suggesting the TAM receptor shedding was affected by these autoimmune diseases. In particular, sMer was increased in SLE, the plasma levels of sMer reflecting disease activity.

## Introduction

Mer is a cell membrane-bound receptor tyrosine kinase (RTK), which together with Axl and Tyro3 constitutes the TAM receptor family [1,2]. The vitamin K-dependent proteins Gas6 (a 'product of growth arrest-specific gene 6') and protein S are important biological ligands for the TAM receptors [3]. Axl is ubiquitously expressed, whereas Tyro3 is predominantly found in the central nervous system and the reproductive system. Mer is named after its expression in monocytes, epithelium and reproductive tissue. Activation of the TAM receptors has been shown to affect a diversity of cellular functions, including survival, proliferation, migration and phagocytosis [4]. Mer is an important mediator of apoptotic cell phagocytosis [5]. It is also important for phagocytosis of photoreceptor outer segments (POS) by retinal pigment epithelial (RPE) cells and genetic defects of the Mer gene *Mertk *are associated with retinitis pigmentosa, which results in the development of blindness [6].

Recent studies have revealed that the TAM receptors have pivotal roles in regulation of innate immunity as regulators of cytokine production in macrophages and dendritic cells [2,7,8]. The TAM receptors also stimulate maturation of natural killer cells. Each of these phenomena depends on cooperative interactions between the TAM receptors and cytokine receptor signaling systems [2]. The importance of the involvement of the TAM receptors in regulation of immunity has been clearly demonstrated in animal models. Thus, mice with triple knockout of the TAM receptors were found to develop severe autoimmune diseases [2]. A milder form of autoimmunity associated with impaired clearance of infused apoptotic cells affected Mer knockout mice. These mice developed progressive lupus-like autoimmunity, with antibodies to chromatin, DNA, and IgG [9].

The TAM receptors are membrane proteins with multiple domains. Two Ig-like and two fibronectin-type III domains constitute the extra-cellular part, which can be proteolytically shed from the cells. A soluble form of the Mer (sMer) receptor tyrosine kinase, comprising the extra-cellular domains, has been found in plasma and shown to inhibit macrophage clearance of apoptotic cells and platelet aggregation [10]. Soluble forms of both Axl and Tyro3 are also present in plasma, at low (subnanomolar) concentrations. The ligand Gas6, which is expressed by many cells, but not much in the liver, is also present at low concentrations in plasma and recently we demonstrated that Gas6 is circulating in complex with sAxl [11]. The plasma concentrations of both Gas6 and sAxl increase in response to acute phase reactions [12]. We have recently found that the plasma concentrations of Gas6 and sAxl correlate to the inflammatory process in systemic lupus erythematosus (SLE) and rheumatoid arthritis (RA) even though both Gas6 and sAxl in most of the patients were within the normal range [13].

Systemic lupus erythematosus (SLE) and rheumatoid arthritis (RA) are autoimmune diseases of unknown etiology [14,15]. SLE patients develop autoantibodies directed against nuclear elements. These autoantibodies form immune complexes that contribute to the disease process. Deposition of these immune deposits in the kidneys initiates an inflammatory response by activating the complement cascade and recruiting inflammatory cells, and the resulting lupus nephritis is a severe complication in SLE.

The possible involvement of the Mer and Tyro3 receptors in human autoimmune diseases has not previously been investigated. In the present study, we have investigated the plasma levels of sMer and sTyro3 in patients with SLE and RA. In addition, we have investigated patients with critical limb ischemia (CLI) as a model disease of non-autoimmune inflammation, because the pathogenesis of CLI does not involve any autoimmune component. CLI is caused by severe atherosclerosis and the inflammatory response is caused by the ischemic tissue damage [16,17]. We now wish to report that sMer is increased in active SLE and to a lesser extent in RA. In contrast, neither sMer nor Tyro3 plasma concentrations are increased in CLI suggesting that the increased sMer in SLE is not the result of the acute phase response.

## Materials and methods

### Patients and controls

Patients with SLE (*n *= 96, 11 men and 85 women) fulfilling four or more ACR classification criteria for SLE [18] were recruited at the Department of Rheumatology, Lund University Hospital, Sweden. The mean age at SLE diagnosis was 35 years and the mean disease duration at the time of the study was 10 years. The disease activity was evaluated using SLEDAI-2K (patients having a mean SLEDAI score of 8.7) and the evaluation time points were selected with the objective of including a wide range of manifestations and SLEDAI scores. As a consequence, the study also included patients without evidence of active disease. The study was approved by the regional ethics board.

Patients with early RA (*n *= 183, 68 men and 115 women) fulfilling the 1958 ARA criteria of definite RA were recruited at the Department of Rheumatology, Lund University Hospital, Sweden between 1985 and 1989. All consecutive patients that were 18 years or older and with symptom duration of less than two years were included. The mean age at diagnosis was 51 years and the mean duration of symptoms was 11 months. The patients were evaluated clinically at least yearly and radiographs of hands and feet were obtained in years 0 to 5 and year 10. The radiographs were evaluated for joint damage according to the Larsen scoring system [19,20]. Analyses of sMer and sTyro3 were performed on plasma samples retrieved within six months of inclusion in the prospective early RA study. The study was approved by the regional ethics board.

Consecutive patients with CLI (*n *= 189) were recruited at the Vascular Centre at Skåne University Hospital, Malmö, Sweden during a 14-month period. This is the referral centre for patients with CLI for 700,000 inhabitants in southern Sweden. The diagnosis of CLI was made according to the Transatlantic Intersociety Consensus (TASC) criteria of ulceration, gangrene or rest pain caused by peripheral artery disease. The characteristics of the patient cohort have been described before [21,22]. The 204 control individuals were drawn from the general population as part of a large prospective study of cardiovascular risk factors and alcohol abuse; none of them had symptomatic CLI, cardiovascular disease, autoimmune diseases or atherosclerosis. The ethics committee at Lund University approved the study and all the patients gave written consent to participate in the study.

### ELISA

The sandwich ELISA kits for sMer (DYC891) or sTyro 3 (DYC5600) were obtained from R&D Systems (Minneapolis, MN, USA). Maxisorp plates (NUNC A/S, Roskilde, Denmark) were coated with the monoclonal antibodies to either hMer or hTyro 3. The plates were blocked with 3% Fish gelatin in 50 mM Tris, 150 mM NaCl, pH 7.4 containing 0.1% Tween 20. The plates were washed five times with phosphate buffered saline (PBS) (137 mM NaCl, 2.7 mM KCl, 8.1 mM Na_2_HPO_4_, 0.1% Tween 20, pH 7.3, 0.2 um filtered) between every step. The EDTA plasma samples were diluted 10 times in the blocking buffer and applied to the plates. The standard curve was made by serial dilution of recombinant protein and negative controls were buffer alone. Biotinylated anti-hMer or anti-hTyro3 antibodies were used for detection together with ABC/HRP complexes according to the manufacturer's instructions. The plates were developed with ortophenylenediamine and hydrogen peroxide and the color reaction was stopped by the addition of 100 ul 1 M sulphuric acid. The absorbance was determined in an automated plate reader at 490 nm. The sMer and sTyro3 ELISAs were not influenced by the presence of autoantibodies such as rheumatoid factor of either IgG, IgM, or IgA class.

### Statistical analyses

Graphpad 4.0 (Graphpad Software, La Jolla, CA, US) was used for all statistical analysis. Correlations were evaluated with Spearman's rank correlation test. Comparisons between groups were performed using the Mann-Whitney U test. A *P *< 0.05 was considered significant for all tests.

## Results

**Figure 1:**
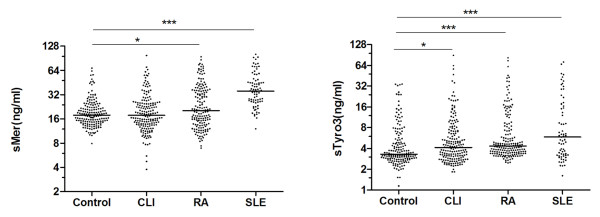
**sMer and sTyro3 in patients with CLI, RA, or SLE**. The concentrations of sMer and sTyro3 were measured using ELISAs and the results were plotted on logarithmic scale (Ln scale). Significances are indicated with the asterisks.

The concentrations of sMer and sTyro3 in controls, as well as in patients, demonstrated skewed distributions (Figure 1). In controls, the median concentration of sMer was 17.8 ng/ml and the range was wide (7.9 to 68.3 ng/ml). There were significantly higher concentrations of sMer both in patients with SLE (median 35.6 ng/ml, range 12.to 101.7; *P *< 0.0001) and in those with RA (median 20.3 ng/ml, range 7.0 to 94.7; *P *= 0.0236), as compared to controls. In particular, the SLE patients demonstrated increased concentrations of sMer, whereas only a sub-group of RA patients demonstrated increased sMer. Patients with CLI demonstrated very similar concentrations and distribution of sMer to controls.

**Figure 2:**
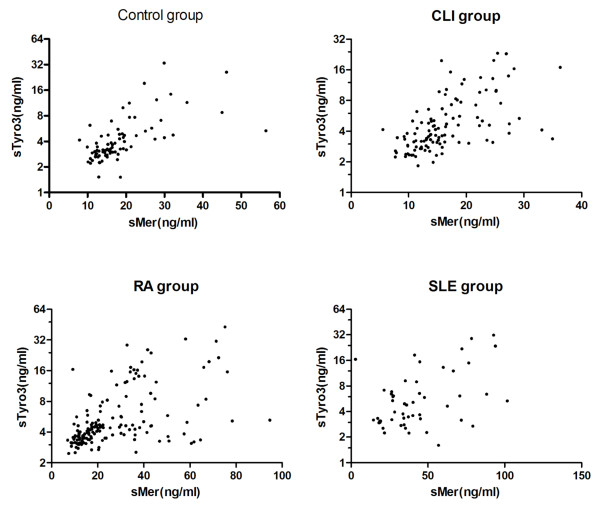
**Correlations between sMer and sTyro3 in the study groups**.

The sTyr3 concentrations were significantly different between controls (median 3.3 ng/ml, range 1.2 to 34.3) and patients with SLE (median 5.84 ng/ml, range1.61 to 71.79), RA (median 4.3 ng/ml, range 2.5 to 82.5) and CLI (median 4.2 ng/ml, range 1.8 to 89) (Figure 1). The sMer and sTyro3 concentrations correlated strongly in all the groups (Figure 2), the Spearman's correlation coefficients were 0.6828 (*P *< 0.0001), 0.648 (*P *< 0.0001), 0.5739 (*P *< 0.001), and 0.3642 (*P *= 0.079) for control-, CLI-, RA-, and SLE-groups, respectively.

**Figure 3:**
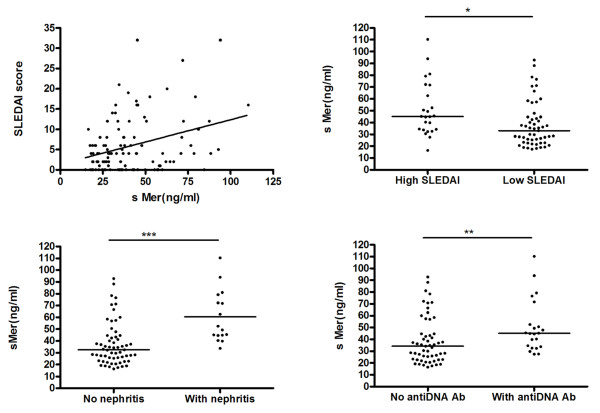
**Correlation between sMer and disease activity in SLE**. sMer concentrations in SLE patients correlate to SLEDAI and are different between patients with or without nephritis and in patients with or without anti-DNA antibodies.

In patients with SLE, the concentrations of sMer demonstrated a weak but significant correlation to the SLEDAI score, (r = 0.25, *P *= 0.0083) (Figure 3). The sMer concentrations in patients (*n *= 24) with a high SLEDAI score (≥ 10) were significantly (*P *= 0.005) higher (45.1 ng/ml, range 16.4 to 110.3) than in those with a low SLEDAI score (< 10) (*n *= 53; 33.0 ng/ml, range 17.4 to 92.8) (Figure 3). The SLEDAI score is composed of many parameters and we, therefore, tested the sMer concentrations against the individual parameters of the SLEDAI score. We found that sMer was particularly correlated to the presence of nephritis (Figure 3). Thus, the patients with nephritis (*n *= 16) had higher sMer (*n *= 16; median 50.9 ng/ml, range 33.8 to 110.3) than those without nephritis (*n *= 61; median 32.6 ng/ml, range 16.4 to 92.8), *P *< 0.0001. This was not caused by decreased glomerular filtration as the sMer levels did not correlate to the serum-creatinine levels.

**Figure 4:**
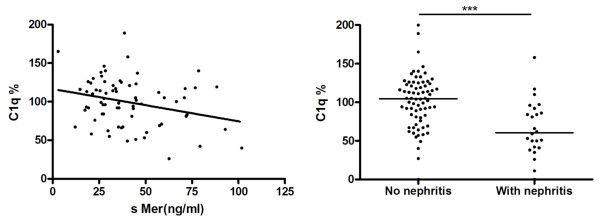
**Negative correlation between sMer and C1q concentrations in SLE patients**.

The concentrations of sMer were also found to correlate to decreasing levels of C1q and the presence of anti-DNA antibodies but not to the presence of rheumatoid factor of IgG, IgM, or IgA class and patients with rheumatoid factor had the same mean sMer and sTyro levels as those without rheumatoid factor. This observation was important because it demonstrated that circulating autoantibodies against immunoglobulins did not disturb the ELISA assays. The correlation between sMer and C1q was negative, r = -0.47, *P *= 0.020 and as expected the nephritis group had significantly (*P *< 0.0001) lower C1q (*n *= 24; median 60.5%, 0 to 158%) than those without nephritis, (*n *= 72; C1q median 104.5%, 0 to 200%) (Figure 4).

The anti-DNA positive SLE patients had higher concentrations of sMer than the negative group; in the positive group (*n *= 22) the median sMer was 45.1 ng/ml (range 27.5 to 110.3) versus 34.3 ng/ml (range 16.4 to 92.8) in the negative group (*n *= 55), (*P *= 0.008) (Figure 3). As expected, there was also a much higher percentage of positivity for anti-DNA antibodies in the nephritis group (70.8%) than in those without nephritis (19.4%).

**Figure 5:**
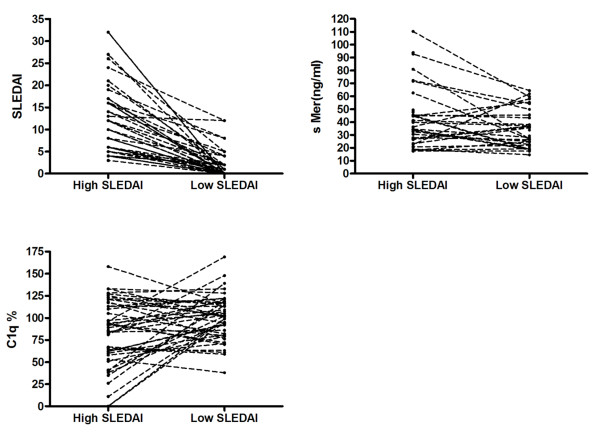
**Parallel changes in SLEDAI, sMer and C1q related to disease activity**. The SLEDAI and sMer concentrations decrease, whereas the C1q levels increase after therapy.

In 45 SLE patients with high SLEDAI (12.0 ± 7.7), samples were also available from time points with low SLEDAI scores (1.9 ± 3.0) (Figure 5); the corresponding concentrations of sMer being 39.9 ng/ml (range 17.40 to 110.3, *n *= 33) and 28.7 ng/ml (range 14.7 to 64.4, *n *= 34), *P *= 0.044, respectively. In contrast, the concentrations of C1q were lower at the time of the higher SLEDAI score, median value 89% (range 0 to 158%, *n *= 45) vs. median value 102% (range 38 to 169%, *n *= 44), *P *= 0.0395, respectively.

In contrast to sMer, the concentrations of sTyro3 were not related to activity parameters like SLEDAI, low C1q or the presence of nephritis.

Although the concentrations of both sMer and sTyro were higher in RA than in controls, there were no correlations to disease parameters such as disease activity as measured by the disease activity score (DAS), the presence and level of rheumatoid factors of IgA, IgM, or IgG class or anti-cyclic citrullinated peptide antibodies (anti-CCP), or elevation of blood sedimentation rate. Furthermore, there were no correlations between sMer or sTyro and radiographic changes in hands and feet determined by the Larsen score at years 0 to 5 and at the 10-year follow-up.

## Discussion

SLE is a chronic autoimmune disease characterized by a plethora of clinical symptoms affecting multiple organs. Patients with SLE have a high tendency to develop autoantibodies against nuclear components. A defective clearance of apoptotic cells is believed to be one of multiple causes of SLE and apoptotic cells are present in the lymph nodes of these patients [23-26]. In addition, isolated macrophages from SLE patients demonstrate reduced ability to phagocytose apoptotic cells [27]. With this background, it is noteworthy that we now demonstrate that patients with SLE have increased concentrations in plasma of sMer, as Mer is an important receptor for phagocytosis of apoptotic cells [28].

The Mer receptor is expressed in macrophages and dendritic cells and is important both for the phagocytosis of the apoptotic cell and also for regulation of the cytokine production of these cells [7,29]. Under normal conditions, the two vitamin K-dependent proteins Gas6 and protein S bind to the negatively charged phospholipid phosphatidylserine that is exposed as part of the apoptotic process. Gas6 and protein S bound to the apoptotic cell surface interact with, and activate, the Mer receptors that are present on the macrophages/dendritic cells. This results in intracellular signaling events, rearrangement of cytoskeleton and engulfment of the apoptotic cell [30,31]. The process requires that the Mer is an intact membrane-spanning protein and depends on activation of the intra-cellular kinase domain of Mer. The presence of fluid phase sMer in the extra-cellular space can compete with cell bound Mer acting as a decoy receptor and, thereby, result in defective phagocytosis [10]. Defective phagocytosis could potentially also be due to increased shedding of Mer receptors from the monocyte/macrophage surface resulting in decreased surface expression of Mer. Whether any of these mechanisms are involved in the defective phagocytic process in SLE remains to be determined.

Normal phagocytosis of apoptotic cells is not associated with inflammation, which is believed to be, at least partly, due to a shift of cytokine production in the phagocytes from pro-inflammatory cytokines to cytokines that are anti-inflammatory [23,32]. Mer is also required for development of B- [33] and T-cell [34,35] tolerance, which is associated with normal phagocytosis of apoptotic cells. The defective clearance of apoptotic cells in SLE contributes to continuous exposure to autoantigens and type-I interferon-1 production by the dendritic cells, which enhance the inflammatory process, the production of autoimmune antibodies and formation of immune-complexes [36,37].

The mechanism through which the plasma concentration of sMer increases in plasma is not clear. Possibly, continuous stimulation by apoptotic debris of the macrophage pool in the SLE patients results in increased Mer expression and shedding. Apoptotic cells have been shown to activate Liver X receptor, which induces expression of Mer [38]. The inflammatory process may also contribute with increased concentrations of metalloproteinases such as tumor necrosis factor alpha-converting enzyme metalloproteinase, which would increase the proteolytic cleavage of the extra-cellular domain of Mer. Cleavage of Mer has also been shown to be enhanced by cell-activating substances such as lipopolysaccharide and phorbol myristate acetate [10].

Although the pathogenesis of SLE has a genetic component, genome wide association studies have not resulted in the identification of predisposing genetic changes [39]. Genetic defects in the *MERTK *gene, demonstrated in humans and in the Royal College of Surgeons (RCS) rat strain, are known to result in defective phagocytosis by the retinal epithelium of the outer membrane of photoreceptors [6]. This causes retinitis pigmentosa, which eventually leads to blindness in the affected individual [40,41]. However, there are no reports suggesting that genetic defects in the *MERTK *gene increase the risk of SLE in humans. The situation is different in mice where a genetically modified Mer receptor (a kinase dead receptor) is demonstrated to develop SLE-like disease [9]. Moreover, the high tendency to develop autoimmune disease in mice lacking the Mer receptor demonstrates the importance of the Mer receptor for regulation of the immune system in association with the apoptotic process.

The pathogenesis of RA has not been associated with defective phagocytosis of apoptotic cells and it is noteworthy that the plasma levels of sMer did not correlate with disease activity [15]. Moreover, in most of the patients, the plasma concentrations of sMer were within the normal range, whereas only very few of the SLE patients had normal sMer levels. This suggests that the sMer concentrations do not reflect the inflammatory process of RA but possibly is related to defective engulfment of apoptotic cells in a subgroup of patients. This conclusion gains further support from the observation that patients with CLI, many of which suffer from severe inflammation, do not have increased plasma levels of sMer. These results stand in contrast to those obtained for Gas6 and sAxl. In many different clinical conditions, including sepsis, CLI and SLE, the concentrations of Gas6 and sAxl demonstrate strong correlation to inflammatory markers suggesting that both Gas6 and Axl expression increases as part of the acute inflammatory response [12,13,42].

The plasma concentrations of sMer were found to correlate with the SLEDAI score in SLE patients suggesting that the sMer concentrations reflect disease activity rather than disease severity. In particular, those patients having nephritis demonstrated increased sMer concentrations, which was not due to decreased clearance as there was no correlation between sMer and creatinin in serum. As expected, the nephritis patients also demonstrated DNA antibodies, decreased concentrations of C1q and a negative correlation between sMer and C1q concentrations. The DNA-containing immune-complexes deposit in the glomerular membrane, which binds C1q and activates a complement thus provoking inflammation and damage to the membrane barrier of the kidney [43]. As demonstrated by immuno-histochemistry of lupus nephritis patients, apoptotic tubular cells positively correlate with mononuclear cell infiltration, which may be recruited by MCP-1, a chemokine for macrophages. SLE patients with nephritis have increased concentrations of MCP-1 in the urine and the MCP-1 concentrations in the urine correlate strongly with proteinuria, hematuria and SLEDAI [44]. Nephritis is a severe complication to SLE and it is crucial to diagnose this complication as early as possible. Possibly, determination of sMer concentration can be helpful in the evaluation of SLE disease activity in general and, in particular, ongoing renal inflammation.

## Conclusions

We demonstrate that the plasma concentrations of the soluble form of the Mer receptor are significantly increased in patients with active SLE. As Mer is important for the phagocytosis of apoptotic cells and regulation of the immune system, the data suggest that shedding of Mer is an active process in the SLE disease process and that the levels of sMer reflect disease activity.

## Abbreviations

ACR: American College of Rheumatology; ARA: American Rheumatology Association; anti-CCP Ab: anti-cyclic citrullinated peptide antibodies; CLI: critical limb ischemia; DAS: Disease Activity Score; MCP-1: monocyte chemotactic protein-1; POS: photoreceptor outer segment; RA: rheumatoid arthritis; RPE: retinal pigment epithelial; RTK: receptor tyrosine kinase; sAxl: soluble Axl receptor; SLE: system lupus erythematosis; SLEDAI: SLE Disease Activity Score; sMer: soluble Mer receptor; sTyro3: soluble Tyro3 receptor; TAM: Tyro3, Axl and Mer.

## Competing interests

The authors declare that they have no competing interests.

## Authors' contributions

WJ carried out the immunoassays, analyzed the data and drafted the manuscript. CE provided reagents, participated in the analysis of experimental data and in the writing of the manuscript. AJ, GS, AAB, AG, BL, EL and TS participated in the design of the study, in the analysis of the patient cohorts, in the statistical analysis and in the writing of the manuscript. BD initiated, planned and supervised the study, participated in the analysis of data and wrote the final manuscript. All authors read and approved the final manuscript.
